# Study on the Effect of Bilateral Mandible Distraction Osteogenesis About the Nutrition Status of Infants With Pierre-Robin Sequence

**DOI:** 10.3389/fped.2021.771333

**Published:** 2021-10-29

**Authors:** Liu Jiayu, Sun Jing, Chen Yiyang, Li Fan

**Affiliations:** ^1^Oral and Maxillofacial Surgery, Guangzhou Women and Children's Medical Center, Guangzhou, China; ^2^Clinical Nutrition Department, Guangzhou Women and Children's Medical Center, Guangzhou, China

**Keywords:** Pierre-Robin Sequence (PRS), distraction osteogenesis (DO), nutrition status, weight for age (WFA), length for age (LFA), blood albumin

## Abstract

**Objective:** By comparing and studying the changes of food intake, weight, body length, BMI, blood albumin level and other indicators of infants with Pierre-Robin Sequence (PRS) before and after Bilateral Mandible Distraction Osteogenesis operation, to explore the effect of distraction osteogenesis on PRS patients about the improvement of nutrition status.

**Methods:** The children with PRS who admitted to the Oral and Maxillofacial Surgery Department of Guangzhou Women and Children's Medical Center from July 2015 to December 2020 were selected. All patients accepted bilateral mandible distraction osteogenesis surgery, and the pre- and post-operative indicators were recorded, such as food intake, weight, length, blood albumin level and others. BMI was calculated based on the indicators mentioned above, and comparative statistical analysis was performed.

**Results:** 1. All patients were fed with whole milk before the first surgery, and the average calorie per kg was 91.8 kcal/kg, significantly lower than the standard (100–150 kcal/kg), suggesting the overall nutritional intake of PRS patients is low; 2. *t* tests for independent samples were used to analyze the pre-operative and post-operative indicators. The WFA percentile increased from 14.16 ± 2.17 to 15.01 ± 1.85% (*P* = 0.0048), WFA *z* score increased from −2.40 ± 0.18 to −1.90 ± 0.14 after the surgery (*P* = 0.0010), LFA percentile increased from 20.04 ± 3.48 to 33.67 ± 4.29% (*P* = 0.0098), LFA z score increased from −2.09 ± 0.19 to −1.42 ± 0.23 (*P* = 0.0009), BMI *z* score increased from −1.95 ± 0.22 to −1.39 ± 0.16 (*P* = 0.0408), ALB raised from 37.06 ± 0.51 to 42.85 ± 0.30 g/L (*P* < 0.001), which indicating that the physique of patients improved after the distraction osteogenesis surgery, mainly was reflected by the lifting of weight and length growth curves; the body shape also improved, indicating that the patients' nutrition status after the surgery is also improved.

**Conclusion:** Bilateral mandible distraction osteogenesis surgery has a positive effect on the nutrition status of children with PRS. This effect is mainly reflected by the improvements of the body physical indicators after surgery.

## Introduction

Pierre-Robin Sequence (PRS) is a group of congenital malformations characterized by micrognathia, glossoptosis, with or without a cleft palate. The patients usually suffer from breathing difficulties and disorders of eating and swallowing due to the small, dysplastic mandible pushing the tongue backward, even attach to the pharyngeal wall, resulting in narrowing of the airway and oropharyngeal cavity, in the newborn and infancy time. Therefore, the growth and development of the patients are often affected, and even could be life threatening in severe cases. There are researches confirming that the nutrition status of patients with PRS without suitable treatment was at the middle-low position compared to the same age group, and the proportion of combination with severe malnutrition is about 53% depending on different statistical methods and populations. Around 50–100% of children with PRS also suffer from varying degree of feeding difficulties, relying on gastric tube, gastrostomy and even parenteral nutrition to sustain living (1, 2). Bilateral mandible distraction osteogenesis (BMDO) surgery has been considered as the first choice for the treatment of severe PRS. After BMDO treatment, the mandible is efficiently lengthened, and the oropharyngeal cavity and airway are enlarged. As a result, difficulties in breathing and eating which are present before surgery are greatly eased (3). According to the above, we reasonably speculate that with the improvement of breathing and eating, the nutrition status and physical level of the patients will also be significantly improved.

In order to analyze the influence of BMDO on the nutrition status of PRS patients, we selected the weight, length, food intake, and blood albumin level of the patients before and after the operation as indicators. We retrospectively analyzed 100 cases of PRS infants who underwent BMDO surgery in our hospital from 2015 to 2020. The changes in the nutritional status before and after surgery furthermore proved the necessity of BMDO surgery for PRS patients, and our result helped us to discuss the choice of surgery indicators from another aspect.

## Materials and Methods

A total of 100 PRS patients treated with BMDO in the Oral and Maxillofacial Surgery Department of Guangzhou Women and Children's Medical Center from 2015.6 to 2020.12 were selected. Their age was between 15 d and14 m before the first surgery. The data and general conditions of the patients are shown in Table 1.

**Table 1:** Baseline characteristics (*n* = 100).

**Gender, *n***	
Male	44
Female	56
Born weight, kg, mean (SD)	2.91 ± 0.53
Gestation, weeks, mean (SD)	38.05 ± 2.15
Admission age, months, mean (SD)	
First	1.28 ± 1.32
Secondary	6.02 ± 2.67
Admission weight, kg, mean (SD)	
First	3.55 ± 0.88
Secondary	6.14 ± 0.92
Admission length, cm, mean (SD)	
First	52.92 ± 3.73
Secondary	63.85 ± 4.48
Admission BMI, mean (SD)	
First	13.06 ± 2.66
Secondary	15.16 ± 1.88

*SD, standard deviation*.

Inclusion criteria of the cases in this study: 1. The diagnosis of PRS is clear, that is, infants with micrognathia, glossoptosis, with or without cleft palate, have not undergone other maxillofacial surgery in the past; 2. General conditions can tolerate surgery under general anesthesia; 3. Distractor implantation and removal operations were completed in our hospital, and the pre- and post-operative data were collected completely.

All patients underwent bilateral mandible distraction osteogenesis under general anesthesia. The incisions were parallel and approximately 1.5 cm away from the lower edge of the mandible near the mandibular angle. The osteotomy lines were designed according to the pre-operative three-dimensional CT model to represent the distraction angle and direction in the simulated operation as much as possible. Distraction started at a speed of 1.2 mm/day after a 48–72 h latency period. In order to avoid excessive movement of the mandible affecting the formation of new bone and the displacement of bone segments, all patients were fed by gastric tube during the distraction period. It takes about 1–2 weeks for the segments to reach the ideal position, when the patients were gradually trained to use a spoon for milk intaking, and the gastric tube was removed when the feeding situation meet patients' daily need. In order to promote the formation and hardening of the new bone and avoid loading of the mandible, all patients' caregivers were told not to use the pacifiers and use spoon for feeding until the removal of the distractor. The physical and biochemical indicators were measured and recorded before two operations, and *t* tests for independent sample were performed to compare the changes of indicators before and after BMDO.

The measurements in this study included weight (kg), body length (cm), milk volume (ml), blood albumin level (g/L), and calculated the related indexes such as Weight for age (WFA), percentile and *z* score, Length for age (LFA), percentile and *z* score, Weight For Length (WFL), percentile and *z* score, BMI and its percentile and *z* scores. All physical indicators were compared with the standard values of normal infants of same age (4). See Table 2 for more details.

**Table 2:** Comparative statistics of independent sample *t* test about physical indicators before and after BMDO.

**Group**		***n***	**Mean**	**Standard deviation mean**	***t***	**Sig**.	**95% Confidence interval**	
							**Lower limit**	**Upper limit**
WFL percentile (%)	1	64	31.4016	3.7554	−0.152	0.5054	−10.8022	9.2574
	2	73	32.1740	3.4220				
WFL *z* score	1	82	−1.1899	0.2481	0.109	0.9141	−0.5638	0.6297
	2	88	−1.2228	0.1721				
WFA percentile (%)	1	65	14.1631	2.1677	−0.297	0.0048**	−6.4456	4.7606
	2	72	15.0056	1.8486				
WFA *z* score	1	98	−2.4022	0.1809	−2.154	0.0010***	−0.9544	−0.0418
	2	91	−1.9042	0.1441				
LFA percentile (%)	1	62	20.0435	3.4772	−2.468	0.0098**	−24.5573	−2.7005
	2	69	33.6725	4.2901				
LFA *z* score	1	82	−2.0851	0.1900	−2.179	0.0009***	−1.2598	−0.0621
	2	88	−1.4242	0.2333				
BMI for age	1	54	18.0259	3.1768	−1.772	0.0789	−15.8501	0.8773
	2	73	25.5123	2.7727				
BMI *z* score	1	82	−1.9450	0.2190	−2.062	0.0408*	−1.0922	−0.0237
	2	88	−1.3870	0.1631				
ALB (g/L)	1	100	37.0550	0.5067	−9.878	0.0000****	−6.9514	−4.6350
	2	110	42.8482	0.2953				

*Group 1: First admission, before BMDO; Group 2: second admission, after BMDO; WFL, weight for length; WFA, weight for age; LFA, length for age; ALB, blood albumin*.

*P < 0.05, **P < 0.01, ***P < 0.001, ****P < 0.0001.

## Results

**Figure 1:**
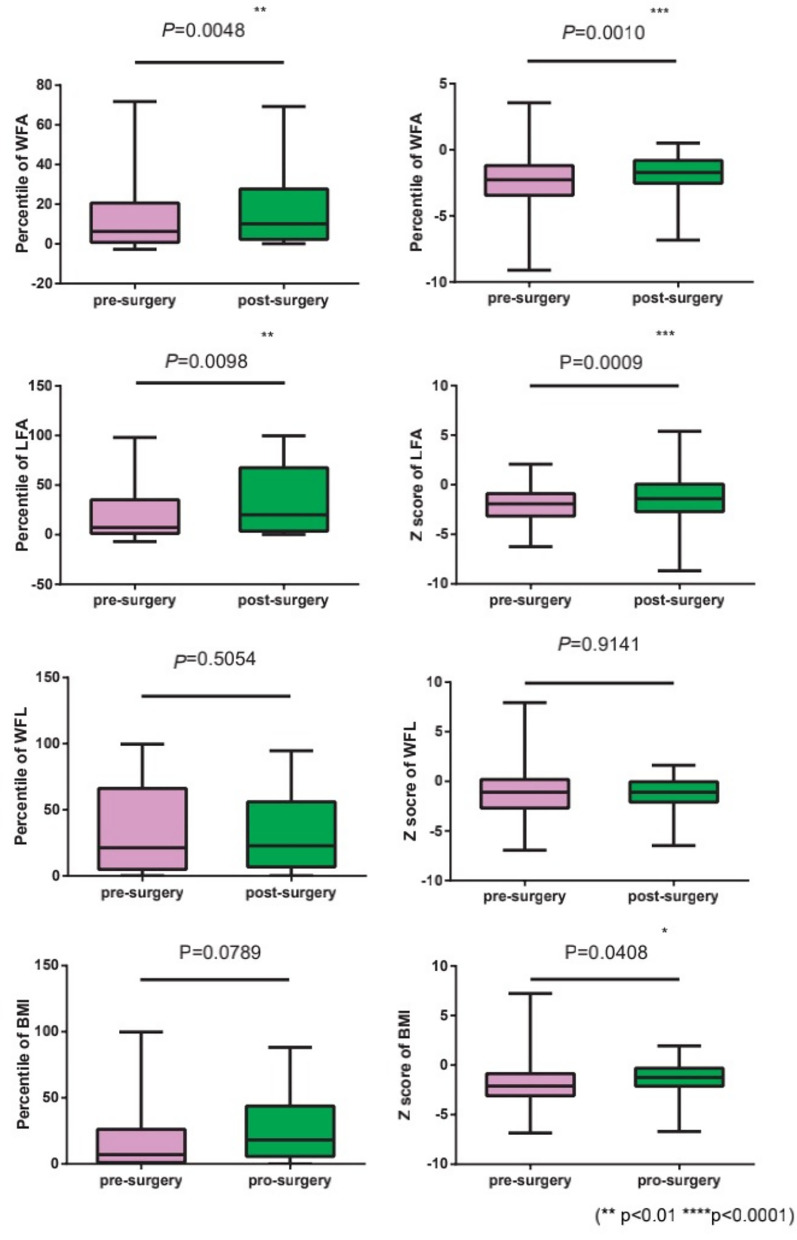
Independent sample comparison statistics of pre- and post-operative body index. *P < 0.05, ***P < 0.001.

**Figure 2:**
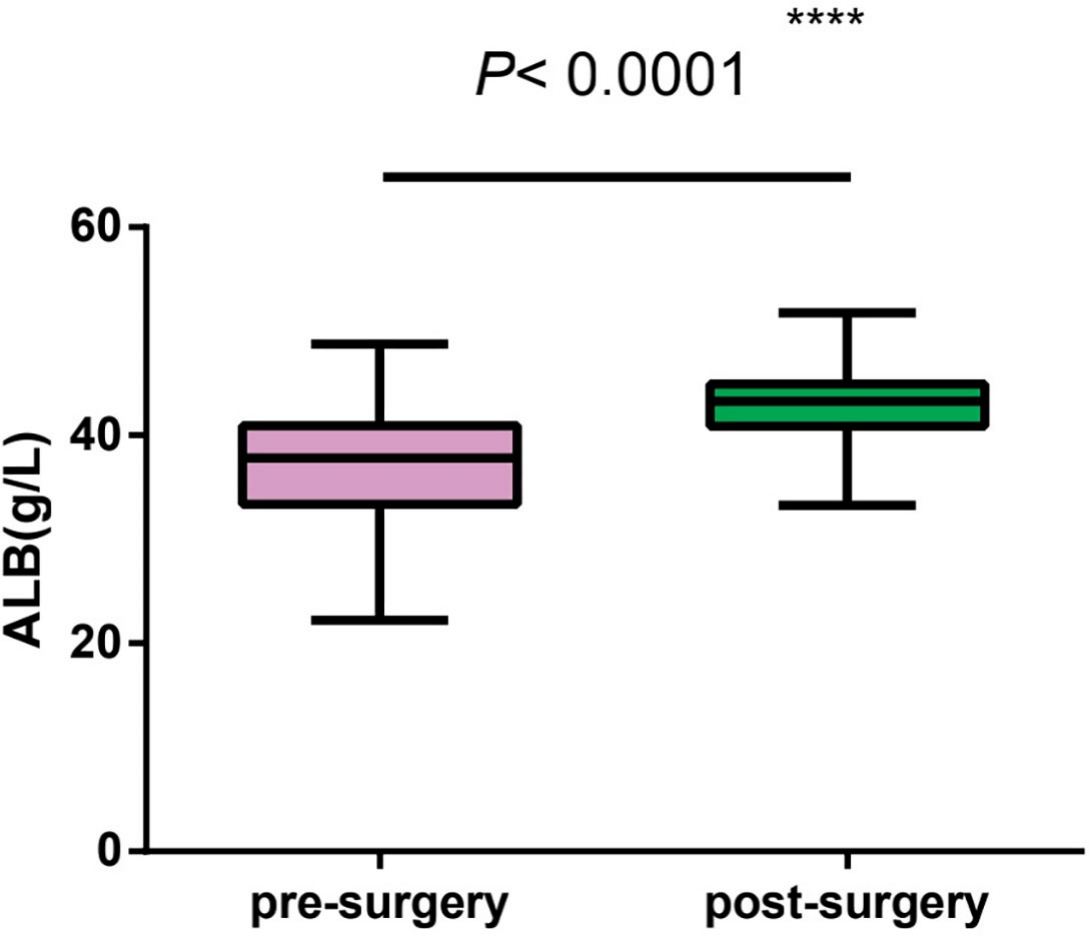
Independent sample comparison statistics of pre- and post-operative blood albumin levels.

1. All patients were fed with total milk before the first surgery. Due to the large age span, there was a big difference in the exact volume of milk intake, so we take the average calorie per kg as the parameter. The average intake was 91.8 kcal /kg, lower than the standard (100~120 kcal/kg) (5) (Table 3).
2. After BMDO, most statistical indicators improved, the WFA percentile increased from 14.16 ± 2.17 to 15.01 ± 1.85% (*P* = 0.0048), WFA *z* score increased from −2.40 ± 0.18 to −1.90 ± 0.14 (*P* =0.0010), LFA percentile increased from 20.04 ± 3.48 to 33.67 ± 4.29% (*P* =0.0098), as the *z* score rose from −2.09 ± 0.19 to −1.42 ± 0.23 (*P* =0.0009). BMI *z* score increased from −1.95 ± 0.22 to −1.39 ± 0.16 (*P* = 0.0408). The changes of indicators above all suggested that the physical levels of patients improved after surgery, especially the body length (Figure 1).
3. Blood albumin level: Before the first surgery, the average blood albumin level in venous blood of all patients was 37.06 ± 0.51 g/L, which was significantly lower than the normal value (40–55 g/L) according to the nutrition status analysis of healthy children in the same age (6). This result indicated that the pre-operative nutrition status was generally poor. The average level of albumin in the venous blood before the second surgery was 42.85 g/L, which was significantly improved from the results before (*P* < 0.0001). Combined with other results of our study, the improvement mentioned above suggested that the body composition (nutrition status) of PRS patients accepted BMDO has improved compared with that before surgery (Figure 2).

**Table 3:** Feeding statistics before BMDO.

	**N**	**Minimum value**	**Maximum value**	**Meverage**	**Standard deviation**
Milk (ml)	93	80.00	900.00	478.3441	180.79093
Milk per kg (ml)	93	25.81	258.06	137.0931	44.45625
Kcal per kg	93	17.29	172.90	91.8524	29.78569

## Discussion

Malnutrition is an imbalance situation between nutritional needs and intake, leading to insufficient accumulation of energy, protein or micronutrients that may have a negative impact on growth, development, and other related results (7). It is one of the common symptoms appeared in patients with PRS. The causes of malnutrition in PRS are various, which may be related to the high energy consumption of respiratory work caused by upper airway obstruction, or to feeding difficulty (FD) caused by micrognathia and glossoptosis (8). The latter one may also be a direct result of respiratory dysfunction. According to the latest research results, some scholars believe that the abnormal development of tongue muscle due to mandible dysplasia is also one of the new reasons worth paying attention to. The incidence of feeding difficulties can be up to 91% in patients with PRS (9), and even if the impact of cleft palate is considered, PRS patients are more likely to have eating and breathing problems than children with ordinary cleft palate (10). According to statistics, 45.8% of patients with PRS need enteral nutritional support (11). Marston et al. (12) reviewed PRS patients who accepted BMDO surgery in 2006, 2009, and 2012 in hospitals across the United States in a 2018 study. Of the 276 children, 17.4% needed gastrostomy to improve food intake, and 16.7% needed the support of total parenteral nutrition. The study by Susarla et al. (1) compared two different treatment methods of PRS, tongue-lip adhesion and distraction osteogenesis, and found that for the children with improved breathing who accepted Tougue-lip Adhesion (TLA), the improvement of swallowing, weight gaining and nutrition status were still not satisfacted; this result may be related to the change of the inherent physiological function structure of the lips and tongue, but it also reflected from another angle that it is not appropriate to only consider the solution of airway obstruction. Whether distraction osteogenesis should be performed or not, the overall nutritional risk of patients should be taken into consideration as well. Although distraction osteogenesis has been considered as an ideal treatment to improve the quality of life and prognosis of PRS patients, its main purpose is still for solving upper airway obstruction. Some doctors still have doubts and questions about the surgery (9) due to the difficulty of surgery, anesthesia, scar and other complications.

According to the author's experience and previous case reports (13). There was no difference in the incidence of post-operative complications for PRS patients accepted surgery in neonatal or non-neonatal period. However, early surgery is conductive to the catch-up of weight, and BMDO within 3 months after birth reduces the need for feeding intervention in PRS patients (14). Resuming oral eating and chewing exercise as soon as possible can also promote the catch-up growth of the mandible (15, 16). Studies have pointed out that repeated airway infections between the age of 2–3 months have a long-term negative impact on growth and development (17). In addition, PRS children accepted BMDO at early age were less likely to have psychomotor development delay and severe malnutrition. Which is also helpful to completing the cleft palate surgery that should be arranged before the age of 1 year, so as to improve their pronunciation (18).

Association and the American Society of Parenteral Nutrition came to a consensus in 2015 (19), and confirmed a set of basic indicators for diagnosing and recording malnutrition in the pediatric population (1 month to 18 years old), recommending energy intake, energy demand and growth indicators to assess nutrition status, generally including length-for-age (LFA), weight-for-age (WFA), and body mass index (BMI) for age. Growth rate is defined as the rate of weight or length/height change over time. This rate of change can be explained as an early sign of a healthy or unhealthy response to the nutritional environment (20). During the growth period, infants need to gain weight at a relatively balanced rate to maintain a relatively stable position on the growth curve, and excessive weight gain and weight loss have been pointed out “relatively independent and more closely related to mortality than other malnutrition indicators (such as BMI for age)” (21). The consensus also pointed out that during the treatment of children, the weight may not change with the changes in body composition at the same time, so the BMI index may not be accurate enough as the only nutritional evaluation index, and the degree of malnutrition may also be underestimated for newborns and small-month-old infants at the first administration to hospitals. Based on previous studies (6, 22), for every 1 g/L decrease in serum albumin concentration, the mortality rate of hospitalized children increased by 137%, and the staying time in the ICU and ward were increased by 28 and 71%, respectively. The blood albumin level can be used as a mature independent indicator for the nutrition status evaluation of children with disease. Therefore, indicators such as height/length and blood albumin level are more suitable for the definition and evaluation of chronic malnutrition (23). This is also why we use LFA, WFA, BMI and blood albumin levels as the evaluation standards in our study.

In previous researches on PRS, researchers have focused more on the improvement of upper airway obstruction and dyspnea symptoms. The study on the nutritional status mainly focused on the changes in weight and body length followed by the airway improvement. More people believed that the improvement of nutritional status is a result of the alleviation of airway problems (24–26). However, some scholars believe that nutritional changes should be studied independently with airway changes, because breathing and swallowing difficulties in PRS patients may not only be related to anatomical variation (such as micrognathia and glossoptosis etc.), but also to abnormal laryngeal development, neurological disorders, and various other associated syndromes (27). For this part of the patients, high-calorie feeding should be applied as soon as possible (28), instead of waiting for the relief of airway symptoms to bring an improvement in nutrition status; the nutritional risk of PRS may not be accompanied by dyspnea, since the symptoms can appear immediately after birth, or may be delayed (29). Previous studies have mostly used to evaluate the changes in weight, length, BMI index pre- and post-operatively without the observation of blood albumin levels' change. Compared with hemoglobin (HGB), serum albumin is relatively less affected by inflammation, blood sampling methods, and has a long half-life period, which is more suitable for defining chronic malnutrition. Our research is the only study to evaluate the changes of serum albumin and body index in PRS patients, and serum albumin levels increased significantly without treatment of albumin transfusion after BMDO, proving that even in some children whose weight gain was not obvious after surgery, the protein nutrition level was also improved. Meanwhile, recovering and maintaining a good nutrition status may accelerate the recovery of airway permeability during growth and development by promoting the development of neuromuscle maturity and coordination (28).

Our research has the following limitations: 1. It is a retrospective study without prospective design. Many physical indicators such as upper arm circumference, blood lipids, blood albumin precursors and others that can further reveal the nutrition status of patients were not included in the routine examinations before surgery; 2, Children with syndromic and non-syndromic PRS were not distinguished and statisted separately, but even in children with syndromic PRS, that is, combined with other neuroendocrine or genetic chromosomal defects, also achieved significant nutritional improvement after BMDO. It suggested that BMDO surgery can help improve PRS patients' food intake in all aspects; 3, There is no control group. Theoretically speaking, the control group should be designed as children with confirmed PRS but without accepting BMDO. However, since PRS may be associated with or without cleft palate that can easily cause eating and swallowing difficulties, it was difficult to distinguish whether the eating difficulties in children without BMDO were related to the micrognathia or the cleft palate. Therefore, this kind of patients were not included in our study. In the follow-up study, prospective design can be carried out to include more evaluation indicators and parameters that affect the nutrition status of PRS patients, such as days of gastric tube use, gastric tube utilization rate and post-operative choking rate, to clarify the relationship between surgery, nutrition and airway.

## Data Availability Statement

The raw data supporting the conclusions of this article will be made available by the authors, without undue reservation.

## Ethics Statement

Ethical review and approval was not required for the study on human participants in accordance with the local legislation and institutional requirements. Written informed consent to participate in this study was provided by the participants' legal guardian/next of kin.

## Author Contributions

LJ and SJ contributed equally to conception and design of the study. LJ organized the database and wrote the first draft of the manuscript. SJ performed the statistical analysis. CY and LF reviewed and revised the manuscript. All authors contributed to the article and approved the submitted version.

## Funding

This work was supported by the Guangzhou City Health Science and Technology Project, Western Medicine-General Guidance Project, 20211A011031.

## Conflict of Interest

The authors declare that the research was conducted in the absence of any commercial or financial relationships that could be construed as a potential conflict of interest.

## Publisher's Note

All claims expressed in this article are solely those of the authors and do not necessarily represent those of their affiliated organizations, or those of the publisher, the editors and the reviewers. Any product that may be evaluated in this article, or claim that may be made by its manufacturer, is not guaranteed or endorsed by the publisher.
